# Phase Error Correction in Time-Averaged 3D Phase Contrast Magnetic Resonance Imaging of the Cerebral Vasculature

**DOI:** 10.1371/journal.pone.0149930

**Published:** 2016-02-24

**Authors:** M. Ethan MacDonald, Nils D. Forkert, G. Bruce Pike, Richard Frayne

**Affiliations:** 1 Departments of Radiology, University of Calgary, Calgary, AB, Canada; 2 Department of Clinical Neuroscience, University of Calgary, Calgary, AB, Canada; 3 Hotchkiss Brain Institute, University of Calgary, Calgary, AB, Canada; 4 Seaman Family MR Research Centre, Foothills Medical Centre, Alberta Health Services, Calgary, AB, Canada; Shenzhen institutes of advanced technology, CHINA

## Abstract

**Purpose:**

Volume flow rate (VFR) measurements based on phase contrast (PC)-magnetic resonance (MR) imaging datasets have spatially varying bias due to eddy current induced phase errors. The purpose of this study was to assess the impact of phase errors in time averaged PC-MR imaging of the cerebral vasculature and explore the effects of three common correction schemes (local bias correction (LBC), local polynomial correction (LPC), and whole brain polynomial correction (WBPC)).

**Methods:**

Measurements of the eddy current induced phase error from a static phantom were first obtained. In thirty healthy human subjects, the methods were then assessed in background tissue to determine if local phase offsets could be removed. Finally, the techniques were used to correct VFR measurements in cerebral vessels and compared statistically.

**Results:**

In the phantom, phase error was measured to be <2.1 ml/s per pixel and the bias was reduced with the correction schemes. In background tissue, the bias was significantly reduced, by 65.6% (LBC), 58.4% (LPC) and 47.7% (WBPC) (*p* < 0.001 across all schemes). Correction did not lead to significantly different VFR measurements in the vessels (*p* = 0.997). In the vessel measurements, the three correction schemes led to flow measurement differences of -0.04 ± 0.05 ml/s, 0.09 ± 0.16 ml/s, and -0.02 ± 0.06 ml/s. Although there was an improvement in background measurements with correction, there was no statistical difference between the three correction schemes (*p* = 0.242 in background and *p* = 0.738 in vessels).

**Conclusions:**

While eddy current induced phase errors can vary between hardware and sequence configurations, our results showed that the impact is small in a typical brain PC-MR protocol and does not have a significant effect on VFR measurements in cerebral vessels.

## Introduction

Phase contrast (PC) magnetic resonance (MR) imaging is a powerful method for measuring blood flow velocity in human subjects and can provide key hemodynamic information about the vasculature. However, concern has been raised about measurement accuracy [1–13], in particular, the impact of phase error from the application of magnetic gradient fields causing a spatially varying offset in the phase across the image [2, 3, 8, 10–12, 14]. This bias can limit the accuracy of quantitative PC-MR measurements. For some regions, like the heart, this phase error has been reported to be as large as 10% to 25% [2, 15]. There are two mechanisms by which the gradients cause phase errors: 1) the concomitant gradient field [14], and 2) gradient-induced eddy currents [2, 3, 15, 16]. These errors characteristically introduce a location-dependent bias into the velocity estimates that can often be observed as non-zero velocity estimates in stationary background tissues [2] that tend to increase with distance from the scanner iso-center [2]. Previous PC-MR cardiac experiments have shown that this error affects velocity measurements and other key hemodynamic metrics, such as volume flow rate (VFR) [2, 8]. On the contrary, it has also been suggested that phase-error correction schemes may actually introduce additional error in the measurements [17].

PC-MR imaging can be performed with either time-varying (*i*.*e*., many phases of the cardiac cycle) or time-averaged (*i*.*e*., a single value averaged over the cardiac cycle). Generally, cardiac studies obtain time-varying PC-MR imaging to compensate for cardiac motion and obtain information about how the flow changes over the cardiac cycle. Although more information is obtained with time-varying imaging it requires significantly more time to acquire to provide the same field of view and acquisition matrix, limiting its clinical application to 2D acquisitions. In the brain where blood is less pulsatile, a time-averaged approach is preferred to obtain higher 3D resolution. Correction for the concomitant gradients [14] is available and automatically performed with most MR systems. Eddy current compensation is typically performed using hardware or software implemented gradient pre-emphasis [18, 19] and any residual eddy current induced phase errors are not usually considered in image reconstruction on the scanner. So far, the majority of work investigating residual phase error correction has been done with single slice, time-varying, cardiac scans, and there has been little work investigating time-averaged 3D phase contrast in the cerebrovascular system, which has prompted this study.

There are several major sources of error when performing PC velocity measurement experiments, including: 1) effects of non-linear gradients [20], 2) partial volume effects [1, 6], 3) concomitant gradient terms [14], and 4) background phase errors from eddy currents. Non-linear gradients and partial volume effects are discussed elsewhere [1, 20]. The other two error sources contribute to an offset in the background phase but originate from fundamentally different mechanisms. The concomitant gradient terms (*i*.*e*., Maxwell terms) can be corrected and removed with processing based on an analytic model prior to assessing phase-offset errors due to eddy currents (such as in this experiment). In this manuscript, when referring to phase error, it is assumed to be eddy current induced phase error unless otherwise specified. Evaluation of and correcting for the residual phase errors due to eddy currents is the focus of this study.

There have been several techniques proposed for correcting eddy current-induced phase errors including: 1) using the local background information in the vicinity of the vessel of interest [3], 2) polynomial fitting to the whole background field [8], 3) *a priori* measurement of the background field with a stationary phantom [21], and 4) using knowledge of gradient trajectory deviation [9]. In this paper, we explore two implementations of the first technique (*i*.*e*., local bias correction, LBC, and local polynomial correction, LPC) and one implementation of the second technique (*i*.*e*., whole brain polynomial correction, WBPC). Each technique has limitations: The local correction techniques presume that the whole bias is due to phase error (in practice, some error could be due to other factors, such as noise), and it also assumes that the bias is the same in the background and the vessel of interest. Fitting to the complete background field can be erroneous near air-tissue interfaces where there is insufficient neighboring signal to estimate the background signal, potentially jeopardizing measurements near the sinuses or in the neck. *A priori* measurement of phase errors in a phantom is imperfect, as eddy currents may vary over time [15]. Measuring the gradient trajectory requires specialized hardware and is limited by the ability to place measurement probes in locations only outside the body.

Most correction methods have been developed especially for cardiac imaging applications, where the phase error may be in the range of 10% to 25% [2, 10, 11, 15]. So far, only a few studies have used these techniques for applications in the time-averaged 3D PC of the brain, which have very different acquisition parameters (field of view, resolution, velocity encoding, etc.). Furthermore, in many older phase-error correction publications targeting the brain, it is likely that no previous correction for the concomitant gradient was applied, as these studies occurred before the concomitant gradient correction was widely adopted [14]. If that correction is not applied first, then the magnitude of errors attributed to eddy currents would be much greater. Finally, even if phase error can be successfully corrected, it is not clear if this correction has a significant influence on VFR measurements of cerebral blood vessels as this has not been thoroughly studied in the head where the flow is different. There are a few reasons why we should expect less phase error in the brain than in cardiac studies: 1) in time-averaged imaging the VFR is generally higher than the diastolic phase of time-varying imaging, and so the error has a lesser relative impact, and 2) the vessels are generally closer to the iso-centre of the magnet and the brain requires a smaller field of view.

In this work, we utilized a typical time-averaged 3D whole brain PC protocol and evaluated the effect of eddy current induced phase-error bias and its correction on VFR measurements using three correction schemes (LBC, LPC, and WBPC). We hypothesized that phase-error correction will have a lesser impact on the VFR in the brain than previous cardiac studies. We evaluated the effects of the three phase-error correction techniques on VFR in a static phantom, in background brain tissue, and in twenty-six vascular segments from thirty human subjects to determine the impact of phase errors and their correction in a typical neurovascular PC acquisition.

## Methods

### Ethics Statement

Permission was obtained from the University of Calgary Ethics Review Board to conduct this study and all subjects provided informed written consent.

### Imaging

Imaging was performed on a 3 T MR scanner (Discovery 750; GE Healthcare, Waukesha, WI). The maximum gradient strength and slew rate of this system are 50 mT m^-1^ and 200 mT m^-1^, respectively. All automatic gradient correction schemes (gradient nonlinearity, concomitant gradient fields, and eddy current pre-emphasis) were calibrated to within the manufacturer specifications. PC imaging was performed using a time-averaged whole-head three-directional Hadamard-encoded PC sequence. The TR/TE/α were 8.3 ms/3.8 ms/10°, the acquisition matrix was 256 × 192 × 192 and the field of view was 22.0 cm × 16.5 cm × 19.2 cm. The receiver bandwidth was ±31.25 kHz. A maximum velocity encoding of 150 cm/s was used in all three directions (anterior-posterior, left-right, superior-inferior; patient coordinate system). Total scan time was 10 min 40 s.

**Fig 1:**
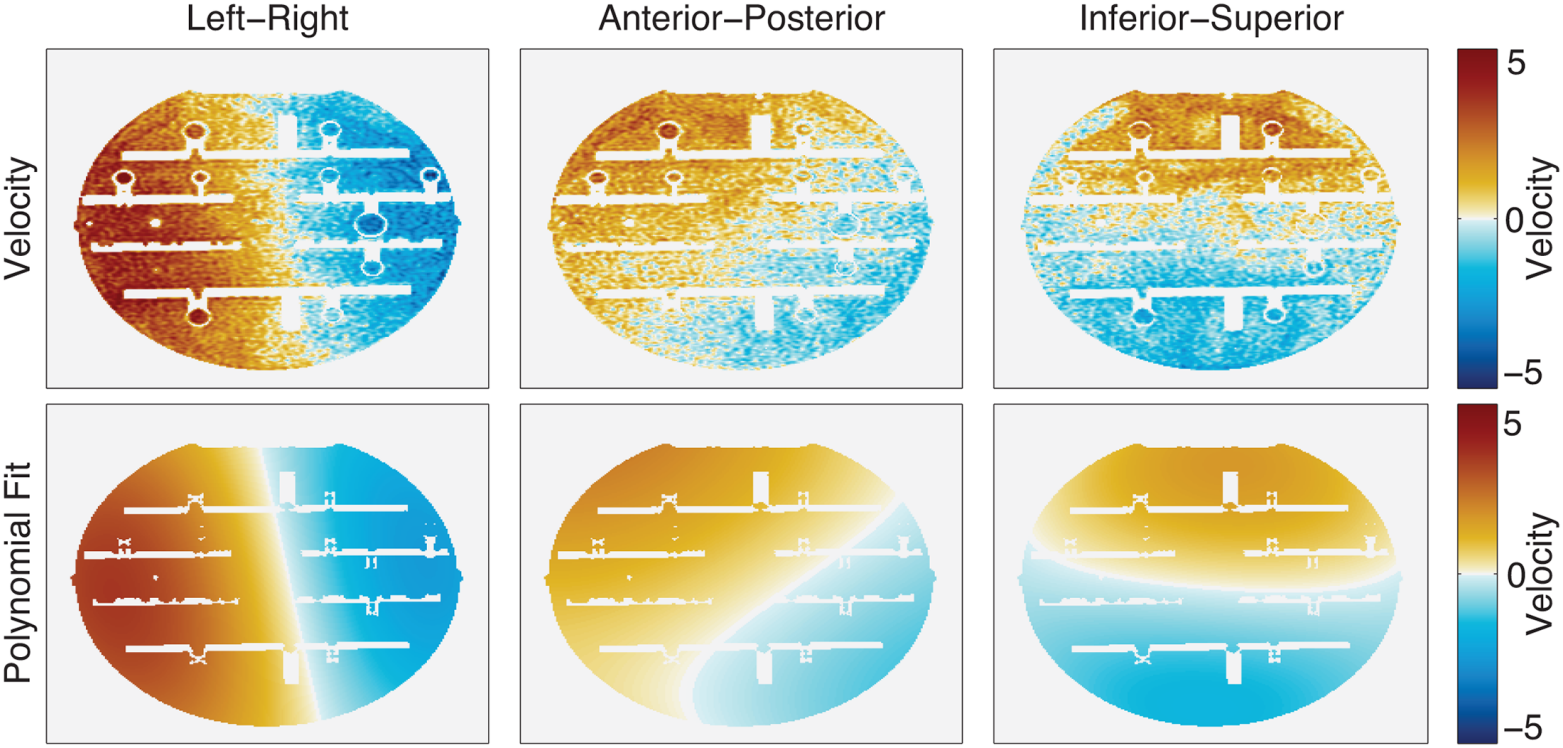
Velocity measurements and whole polynomial fit in an axial slice of a static phantom. The top row shows the measured velocity in the phantom and the bottom row shows the whole brain polynomial fit. Measurements corresponding to these images can be found in Table 1.

PC-MR imaging was first performed on a static phantom (Magphan^®^ Quantitative Imaging Phantom, made by the Phantom Laboratory, Salem NY) to assess the phase offset in the background (Fig 1). This phantom was selected to represent the size of a human head. Although there is no flow in a static phantom, the background will still have some bias from eddy current induced phase errors.

Imaging was performed in 30 healthy human subjects (13 females, aged between 18 and 64 years of age, mean age 33 years and standard deviation 9 years). This cohort was described in a previously published study [22]. The imaging volume was positioned to cover the brain and cerebral vasculature from just below the carotid bifurcation to top of the head. Vendor-provided, post-acquisition, corrections for gradient nonlinearities and Maxwell terms were automatically applied prior to this analysis [14].

### Measurements

**Fig 2:**
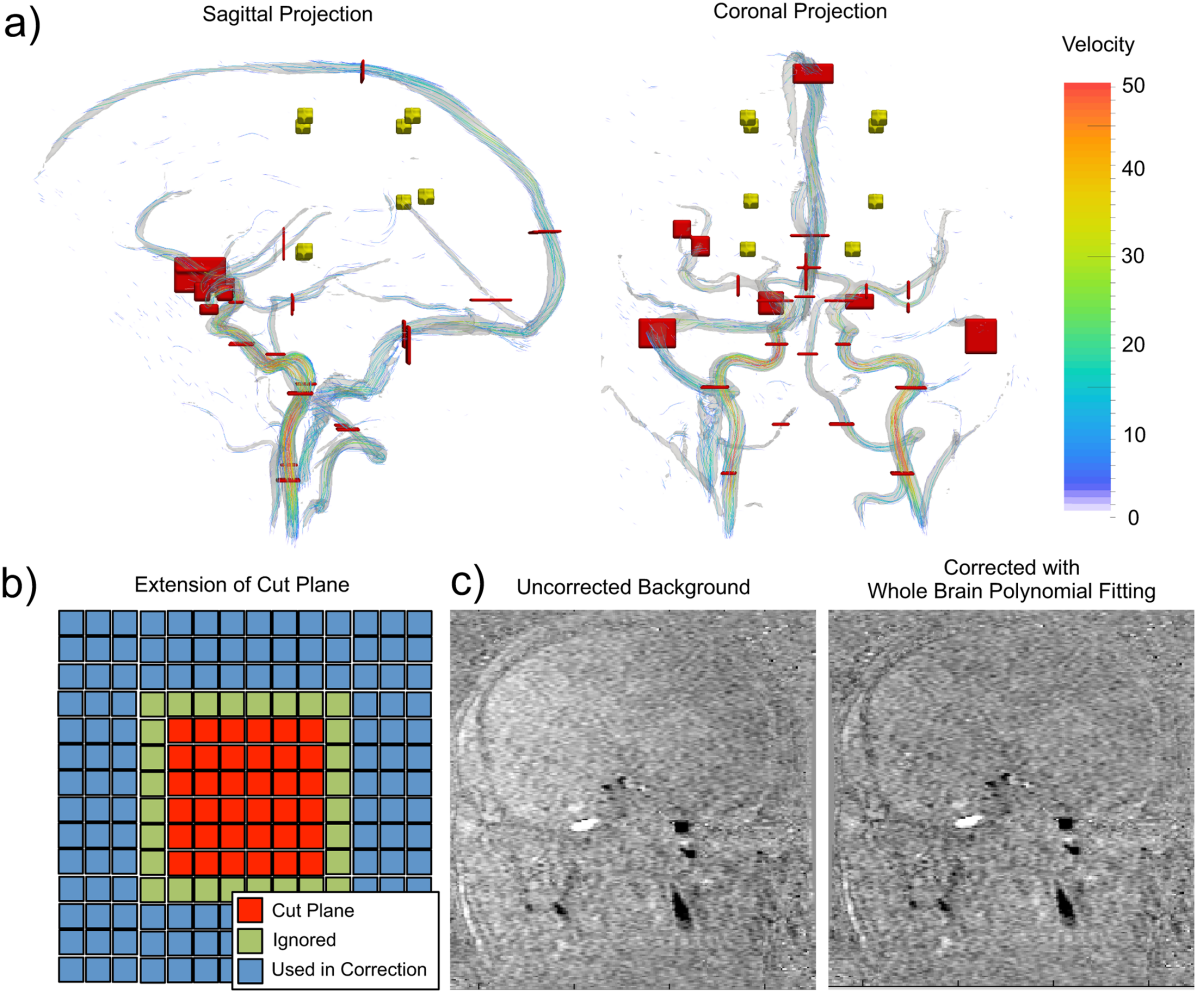
Methodology overview: a) Cerebrovascular phase contrast (PC) angiographic rendering with background cut-planes (yellow) and vessel cut-planes (red). Background cut-planes were placed at the three orthogonal orientations (anterior-posterior, left-right, inferior-superior). Vessel cut-planes were placed approximately orthogonal to the local vessel midline. b) Schematic of pixel selection local VFR estimation (red pixels) and for calculation of bias correction and local polynomial corrections (blue pixels). c) Uncorrected background (left image) and the same data after whole brain polynomial background field correction (right image) showing reduction of background phase error.

Phase error of the background was measured by placing cut-planes (*i*.*e*., planes used to integrate the velocity flux, Fig 2), at 8 different locations and in 3 orientations in the phantom and the brain tissue of each subject (*i*.*e*., 24 cut-planes per subject). The background cut-planes (size of 6 × 6 pixels) were positioned in regions without visible vasculature so that we could assume that the tissue was stationary (net velocity was zero cm/s or VFR was zero (ml/s)/pixel). Background tissue cut-planes were located at eight pre-specified locations in the phantom and brain tissue (Fig 2a, yellow planes), typically, four to eight centimeters from the magnet iso-centre. An experienced operator adjusted the cut-plane locations manually in each subject to avoid vessels. At each location, three cut-planes were defined perpendicular to the anterior-posterior, left-right, and superior-inferior orientations. The volume flow rate (VFR) per pixel was calculated from the cut-plane by integrating the velocity and multiplying by the pixel area [22] and dividing by the number of pixels. The value was normalized by the number of pixels to account for different sized areas of vessel segments in later sections. A total of 24 measurements were taken in the phantom, and 720 background tissue measurements were collected across the 30 subjects.

Vessel cut-planes were placed across 26 major cerebrovascular segments following the procedure detailed in [22]. Vessel cut-planes were made at four locations along the internal carotid arteries (ICA), along the vertebral arteries, along the middle cerebral artery (MCA) M1 segment, and in the M2 segments, across anterior communicating artery (ACom), along the anterior cerebral arteries (ACA), along the basilar artery, and across the posterior cerebral arteries (PCA), along the sagittal sinus, vein of Galen, and transverse sinuses (Fig 2a, red cut-planes). These cut-planes were of the size required to measure the VFR through the vessel segment so that the size was set uniquely for each vessel. Only pixels in the vessel with velocity >7 cm/s were used in the calculation (the value of 7 cm/s was predetermined as a threshold that would segment the vessels from the background noise and background phase offset).

### Correction Techniques

Three methods were used to correct for the eddy current-induced phase error: 1) local bias correction (LBC), 2) local polynomial correction (LPC), and 3) whole brain polynomial correction (WBPC). With the first two methods correction was performed by extending each cut-plane and using the area in the extended region to estimate the local background offset (Fig 2b). This was done in both the phantom and *in vivo* datasets. The cut-plane was first dilated by one pixel. This first ring was not used for background-offset estimation to prevent erroneous results due to partial volume effects. A further dilation of the cut-plane by three pixels was used to define a second ring to be used for local background-offset estimation. Although there are a number of approaches to define the local region prior to estimating the phase error, this approach was selected because it is similar to a previous work [3]. Pixels in the second ring with velocity >7 cm/s were not used for bias estimation in order to exclude possible adjacent vessels. This exclusion was particularly important above the carotid bifurcation to exclude the external carotid artery. For bias correction, the mean velocity of the second ring area was calculated as an estimate of the phase error and subtracted from velocity estimates in the cut-plane measurement (which was used to calculate the VFR in the inner region of that cut-plane). For the LPC technique, the phase bias in the second ring were fit (with least squares) to a second-order polynomial to estimate the phase error within the inner region of the cut plane, and then the estimated bias was subtracted.

For the third technique, WBPC, masks of the brain tissue and vessels were created with fixed thresholds (>10% of the maximum magnitude signal to segment the brain tissue and vessels and >7 cm/s to select vessels) to isolate the background by excluding the vessel segmentation mask from the brain tissue segmentation mask. The thresholds used here were selected based on a previous study [22] to accurately segment the background and vessels. The stationary background was then fit to a third-order polynomial function using the least squares method. The resulting function was then used to estimate and correct the phase error across the brain, including vessels. Fig 1 row 2 and Fig 2c shows an example of the impact of this correction.

### Statistics

A Kolmogorov-Smirnov test was used to confirm normality of the data with 95% confidence. A six-way ANOVA test was performed on the background tissue measurements comparing the effect of subject, correction scheme, measurement location (three factors: left/right, anterior/posterior, and inferior/superior), and cut-plane orientation. An additional three-way ANOVA test (factors: subject, vessel, and correction scheme) was performed on the vessel measurements to test for significant effects in phase error correction. Where appropriate mean ± standard deviation values were reported. A level of *p* < 0.05 was considered significant. Corrected vessel measurements were correlated with uncorrected measurements and the difference in uncorrected and corrected measurements was assessed with a Bland-Altman analysis [23].

## Results

Fig 1 shows the eddy current induced phase error in a static phantom, and in Table 1 measurements from the 24 cut planes placed around the iso-center at different orientations are reported. Although the phantom has no flow, there is an observable bias in the velocity across it. The bias exists in all encoding directions, but it can be seen that it is largest in the left-right direction (the phase encoding direction). The whole brain polynomial fitting is applied in the second row of the image, and additional noise can be seen when comparing the first and second rows. VFR per pixel was measured to be less than 2.1 (ml/s)/pixel. The correction schemes lowered the range to <0.6 (ml/s)/pixel, <0.3 (ml/s)/pixel, and <0.5 (ml/s)/pixel, for the LBC, LPC and WBPC. Although the general trend was a reduction in the bias, in some cases the magnitude of the phase error was found to be larger with the correction.

**Table 1:** Absolute volume flow rate (VFR) for cut-planes placed of static phantom in Fig 1. As each cut-plane was placed in stationary background, a VFR of zero (ml/s)/pixel was expected in the measurements. A larger reduction in VFR estimates indicates better calibration performance.

Location	Absolute Volume Flow Rate [(ml/s)/pixel]			
	Uncorrected	LBC	LPC	WBPC
Cut-Plane Orientation Anterior-Posterior				
Inferior-Anterior-Left	0.534	-0.116	-0.070	-0.022
Inferior-Anterior-Right	-0.032	0.031	0.068	-0.042
Inferior-Posterior-Left	0.068	-0.122	-0.081	0.042
Inferior-Posterior-Right	-0.847	-0.145	-0.226	-0.392
Superior-Anterior-Left	0.981	-0.009	-0.022	0.098
Superior-Anterior-Right	0.170	0.020	-0.033	-0.028
Superior-Posterior-Left	-0.135	-0.234	-0.171	-0.409
Superior-Posterior-Right	-0.470	-0.046	-0.002	-0.124
Cut-Plane Orientation Left-Right				
Anterior-Inferior-Left	1.116	0.384	0.236	0.100
Inferior-Anterior-Right	1.465	0.151	0.204	0.076
Inferior-Posterior-Left	-1.341	-0.418	-0.125	-0.078
Inferior-Posterior-Right	-1.111	-0.048	0.157	-0.205
Superior-Anterior-Left	1.235	0.307	-0.046	-0.015
Superior-Anterior-Right	1.830	0.124	-0.019	0.224
Superior-Posterior-Left	-1.195	-0.299	-0.182	-0.201
Superior-Posterior-Right	-0.743	-0.144	0.005	-0.090
Cut-Plane Orientation Inferior-Superior				
Inferior-Anterior-Left	1.705	0.439	0.238	-0.053
Inferior-Anterior-Right	0.795	0.088	0.012	0.070
Inferior-Posterior-Left	2.090	0.592	0.261	0.183
Inferior-Posterior-Right	1.050	0.094	-0.041	0.209
Superior-Anterior-Left	0.604	0.155	0.003	0.083
Superior-Anterior-Right	-0.569	0.024	0.151	-0.067
Superior-Posterior-Left	0.646	0.064	0.028	0.009
Superior-Posterior-Right	-0.464	0.140	0.155	-0.045

LBC—local bias correction, LPC—local polynomial correction, WBPC—whole brain polynomial correction

**Fig 3:**
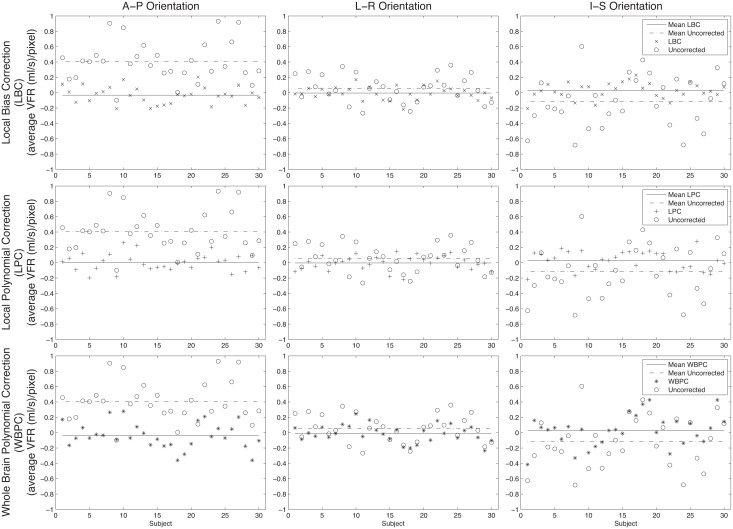
Correction performance in background tissue measurements for a single location (Inferior-Anterior-Left). The columns represent different cut-plane orientations and the rows correspond to the different correction methods. The open circles represent the uncorrected measurements and the symbols represent the results after applying the three correction techniques. The solid black lines show the average correction and the dashed lines represent the average uncorrected measurements versus subject. With all orientations and methods the corrected mean measurements in stationary tissue were closer to zero (ml/s)/pixel.

Fig 3 shows how the *in vivo* measurements at one background location (Inferior-Anterior-Left location), had errors that varied with orientation for a given location, but were generally reduced towards zero ml/s as expected. The mean uncorrected error at this location across the 30 subjects was small (0.26 (ml/s)/pixel) and after correction approached zero ml/s (0.07 (ml/s)/pixel for LBC, 0.09 (ml/s)/pixel for LPC, and 0.11 (ml/s)/pixel for WBPC). Similar trends were observed in the seven other background tissue cut-planes (Tables 2 and 3). The majority of the observed error was removed from measurements in background tissue, independent of the correction scheme used (Table 3). The average absolute VFR reduction after correction across the 24 cut planes was 57.2% ± 20.6% (65.6% ± 14.5% for LBC, 58.4% ± 20.5% for LPC and 47.7% ± 22.5% for WBPC, Table 3). The LBC scheme had the lowest average corrected mean VFR (0.07 ± 0.18 (ml/s)/pixel, Table 2) and had the best performance over this data-set, compared to the two other correction schemes. The Kolmogorov-Smirnov test confirmed normality of the background tissue measurements with 95% confidence. Among the background tissue measurements, the ANOVA test revealed statistically significant effects for the correction scheme (*p* < 0.001), left-right location (*p* = 0.015), and orientation (*p* < 0.001). Subject (*p* = 0.074), or anterior/posterior (*p* = 0.817) and inferior/posterior (*p* = 0.457) measurement location were not significant. A post-hoc one-way ANOVA test, however, found no significant differences between the three correction schemes (*p* = 0.242) across all locations and orientations.

**Table 2:** Absolute volume flow rate (VFR) for cut-planes placed in background tissue. These numbers represent the absolute measured VFR before and after correction. Measurements for each location and orientation are averaged over the thirty subjects. Population mean and standard deviation are provided. As each cut-plane was placed in stationary background tissue, a VFR of zero (ml/s)/pixel was expected in the measurements. A larger reduction in VFR estimates indicates better calibration performance.

Location	Absolute Volume Flow Rate [(ml/s)/pixel]			
	Uncorrected	LBC	LPC	WBPC
Cut-plane Orientation Anterior-Posterior				
Inferior-Anterior-Left	0.414 ± 0.250	0.092 ± 0.068	0.089 ± 0.067	0.139 ± 0.096
Inferior-Anterior-Right	0.154 ± 0.103	0.065 ± 0.051	0.073 ± 0.054	0.090 ± 0.067
Inferior-Posterior-Left	0.292 ± 0.193	0.085 ± 0.063	0.108 ± 0.061	0.161 ± 0.130
Inferior-Posterior-Right	0.356 ± 0.218	0.114 ± 0.069	0.110 ± 0.082	0.149 ± 0.103
Superior-Anterior-Left	0.410 ± 0.153	0.061 ± 0.047	0.075 ± 0.062	0.095 ± 0.051
Superior-Anterior-Right	0.249 ± 0.218	0.095 ± 0.082	0.102 ± 0.091	0.184 ± 0.134
Superior-Posterior-Left	0.201 ± 0.192	0.086 ± 0.071	0.093 ± 0.076	0.153 ± 0.121
Superior-Posterior-Right	0.455 ± 0.156	0.048 ± 0.039	0.052 ± 0.043	0.078 ± 0.055
**Average**	**0.316 ± 0.109**	**0.081 ± 0.021**	**0.088 ± 0.020**	**0.131 ± 0.038**
Cut-plane Orientation Left-Right				
Inferior-Anterior-Left	0.188 ± 0.176	0.093 ± 0.079	0.121 ± 0.093	0.168 ± 0.134
Inferior-Anterior-Right	0.283 ± 0.180	0.091 ± 0.067	0.080 ± 0.064	0.103 ± 0.092
Inferior-Posterior-Left	0.149 ± 0.101	0.055 ± 0.041	0.070 ± 0.044	0.076 ± 0.047
Inferior-Posterior-Right	0.206 ± 0.165	0.092 ± 0.071	0.092 ± 0.069	0.134 ± 0.095
Superior-Anterior-Left	0.194 ± 0.119	0.065 ± 0.059	0.076 ± 0.057	0.136 ± 0.105
Superior-Anterior-Right	0.478 ± 0.121	0.070 ± 0.069	0.082 ± 0.070	0.069 ± 0.063
Superior-Posterior-Left	0.139 ± 0.154	0.083 ± 0.060	0.110 ± 0.082	0.119 ± 0.103
Superior-Posterior-Right	0.359 ± 0.151	0.073 ± 0.046	0.093 ± 0.055	0.095 ± 0.064
**Average**	**0.250 ± 0.117**	**0.078 ± 0.014**	**0.091 ± 0.017**	**0.113 ± 0.033**
Cut-plane Orientation Inferior-Superior				
Inferior-Anterior-Left	0.129 ± 0.088	0.060 ± 0.040	0.068 ± 0.046	0.061 ± 0.057
Inferior-Anterior-Right	0.206 ± 0.152	0.073 ± 0.061	0.094 ± 0.070	0.104 ± 0.080
Inferior-Posterior-Left	0.132 ± 0.105	0.062 ± 0.055	0.102 ± 0.068	0.100 ± 0.069
Inferior-Posterior-Right	0.504 ± 0.147	0.040 ± 0.028	0.049 ± 0.040	0.097 ± 0.050
Superior-Anterior-Left	0.128 ± 0.093	0.080 ± 0.062	0.106 ± 0.071	0.096 ± 0.077
Superior-Anterior-Right	0.253 ± 0.157	0.058 ± 0.051	0.075 ± 0.050	0.103 ± 0.088
Superior-Posterior-Left	0.145 ± 0.112	0.061 ± 0.047	0.061 ± 0.040	0.082 ± 0.063
**Average**	**0.208 ± 0.128**	**0.062 ± 0.012**	**0.082 ± 0.021**	**0.095 ± 0.017**
**Overall Average**	**0.258 ± 0.122**	**0.074 ± 0.018**	**0.087 ± 0.019**	**0.113 ± 0.033**

LBC—local bias correction, LPC—local polynomial correction, WBPC—whole brain polynomial correction

**Table 3:** Absolute VFR reduction as a percentage of the uncorrected VFR for each of the three correction schemes: local bias correction (LBC), local polynomial correction (LPC), whole brain polynomial correction (WBPC) for cut-planes placed in background tissue as describe in Table 1. Group (by cut-plane orientation, defined in Fig 2) and overall mean and standard deviation are provided. A larger absolute VFR reduction indicates better calibration performance in stationary tissue.

Location	Absolute VFR Reduction (%)		
	LBC	LPC	WBPC
Cut-plane Orientation Anterior-Posterior			
Inferior-Anterior-Left	77.8%	78.5%	66.4%
Inferior-Anterior-Right	57.8%	52.6%	41.6%
Inferior-Posterior-Left	70.9%	63.0%	44.9%
Inferior-Posterior-Right	68.0%	69.1%	58.1%
Superior-Anterior-Left	85.1%	81.7%	76.8%
Superior-Anterior-Right	61.8%	59.0%	26.1%
Superior-Posterior-Left	57.2%	53.7%	23.9%
Superior-Posterior-Right	89.5%	88.6%	82.9%
**Average**	**71.0% ± 12.2%**	**68.3% ± 13.5%**	**52.6% ± 22.1%**
Cut-plane Orientation Left-Right			
Inferior-Anterior-Left	50.5%	35.6%	10.6%
Inferior-Anterior-Right	67.8%	71.7%	63.6%
Inferior-Posterior-Left	63.1%	53.0%	49.0%
Inferior-Posterior-Right	55.3%	55.3%	35.0%
Superior-Anterior-Left	66.5%	60.8%	29.9%
Superior-Anterior-Right	85.4%	82.8%	85.6%
Superior-Posterior-Left	40.3%	20.9%	14.4%
Superior-Posterior-Right	79.7%	74.1%	73.5%
**Average**	**63.6% ± 14.8%**	**56.8% ± 20.6%**	**45.2% ± 27.4%**
Cut-plane Orientation Inferior-Superior			
Inferior-Anterior-Left	53.5%	47.3%	52.7%
Inferior-Anterior-Right	64.6%	54.4%	49.5%
Inferior-Posterior-Left	53.0%	22.7%	24.2%
Inferior-Posterior-Right	92.1%	90.3%	80.8%
Superior-Anterior-Left	37.5%	17.2%	25.0%
Superior-Anterior-Right	77.1%	70.4%	59.3%
Superior-Posterior-Left	57.9%	57.9%	43.4%
**Average**	**62.2% ± 16.5%**	**50.1% ± 24.0%**	**45.4% ± 19.5%**
**Overall Average**	**65.6% ± 14.5%**	**58.4% ± 20.5%**	**47.7% ± 22.5%**

LBC—local bias correction, LPC—local polynomial correction, WBPC—whole brain polynomial correction

**Fig 4:**
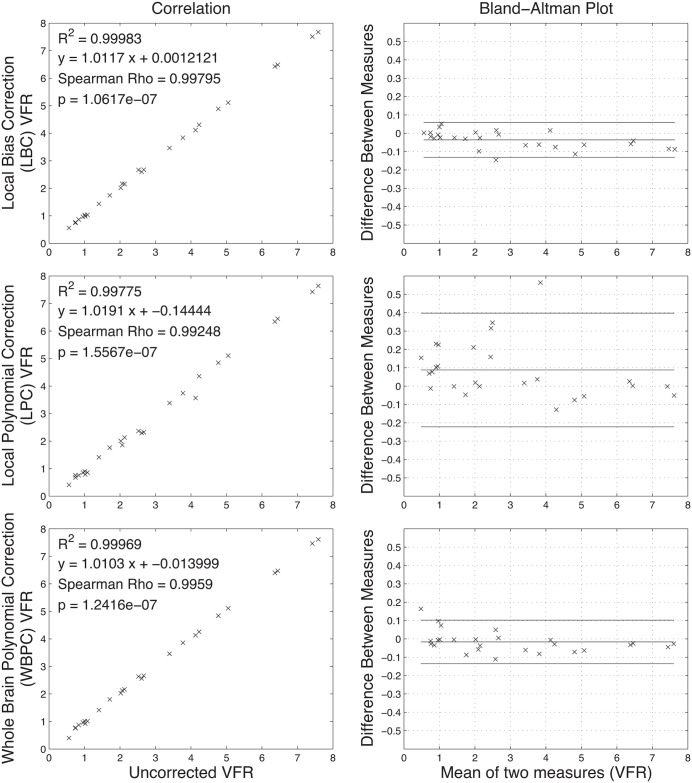
Correlation (left column) and Bland-Altman (right column) plots. Each row shows the results for a different correction scheme. A strong correlation was observed with each correction method. The Bland-Altman graphs plot the mean difference and two standard deviations of the difference. The average of the differences for LBC, LPC, and WBPC methods were found to be -0.036 ± 0.048 ml/s (mean ± standard deviation), 0.088 ± 0.155 ml/s, and -0.017 ± 0.059 ml/s, respectively.

The vessel VFR measurements also changed with the correction scheme (Table 4). The average change of the VFR in the vessel segments, however, was small (1.04% for LBC, -6.04% for LPC, and -0.51% for WBFC). The average coefficient of variation remained essentially unchanged from 0.42 in the case of the uncorrected measurements to 0.46, 0.45, and 0.46 for LBC, LPC and WBPC schemes, respectively. The corrected measurements were strongly correlated with the uncorrected measurements (Fig 4). In the Bland-Altman plots, it can be seen that the mean impact of correction in these measurements was small: -0.04 ± 0.05 ml/s for LBC, 0.09 ± 0.16 ml/s for LPC, and -0.02 ± 0.06 ml/s for WBPC; no pre/post correction differences were significant (*p* = 0.997). Again, there was no significant difference between the different correction schemes (*p* = 0.738).

**Table 4:** Volume flow rate (VFR) for cut-planes placed across twenty-six cerebral vessels showing uncorrected, and LBC, LPC and WBPC corrected estimates. Overall none of the correction schemes had a significant impact on VFR (*p* = 0.997).

	Volume Flow Rate [ml/s]			
Location	Uncorrected	LBC	LPC	WBPC
Right Distal to Bifurcation ICA	6.446 ± 1.787	6.487 ± 1.733	6.445 ± 1.683	6.471 ± 1.792
Left Distal to Bifurcation ICA	7.587 ± 1.687	7.675 ± 1.684	7.639 ± 1.687	7.614 ± 1.692
Right Cervical ICA	6.365 ± 1.478	6.424 ± 1.475	6.340 ± 1.463	6.398 ± 1.486
Left Cervical ICA	7.421 ± 1.832	7.506 ± 1.819	7.423 ± 1.794	7.465 ± 1.818
Right Cavernous ICA	4.771 ± 1.586	4.885 ± 1.665	4.847 ± 1.544	4.842 ± 1.614
Left Cavernous ICA	5.048 ± 1.478	5.111 ± 1.484	5.103 ± 1.501	5.111 ± 1.486
Right Supraclinoid ICA	3.398 ± 0.876	3.463 ± 0.863	3.381 ± 0.823	3.459 ± 0.885
Left Supraclinoid ICA	3.776 ± 1.066	3.838 ± 1.061	3.739 ± 1.076	3.857 ± 1.061
Right Vertebral	1.409 ± 0.912	1.434 ± 0.929	1.411 ± 0.915	1.413 ± 0.905
Left Vertebral	2.022 ± 0.940	2.018 ± 0.891	2.004 ± 0.904	2.025 ± 0.930
Basilar	2.129 ± 0.752	2.154 ± 0.736	2.131 ± 0.772	2.166 ± 0.766
Right PCA	1.084 ± 0.454	1.033 ± 0.460	0.859 ± 0.355	1.011 ± 0.410
Left PCA	1.019 ± 0.337	0.985 ± 0.345	0.789 ± 0.256	0.922 ± 0.291
Right MCA M1	2.067 ± 0.648	2.165 ± 0.650	1.856 ± 0.560	2.124 ± 0.657
Right MCA Superior M2	0.838 ± 0.502	0.865 ± 0.965	0.760 ± 0.834	0.872 ± 0.947
Right MCA Inferior M2	0.746 ± 0.391	0.764 ± 0.813	0.678 ± 0.713	0.773 ± 0.811
Left MCA M1	2.669 ± 0.797	2.676 ± 0.799	2.324 ± 0.689	2.664 ± 0.794
Left MCA Superior M2	1.008 ± 0.611	1.032 ± 0.625	0.900 ± 0.517	1.012 ± 0.593
Left MCA Inferior M2	0.957 ± 0.395	0.965 ± 0.409	0.855 ± 0.361	0.964 ± 0.400
ACA	1.714 ± 0.679	1.744 ± 0.664	1.761 ± 0.646	1.801 ± 0.685
Acom	0.563 ± 0.579	0.561 ± 0.575	0.409 ± 0.463	0.399 ± 0.502
Mid Superior Sagittal Sinus	2.612 ± 0.914	2.596 ± 0.917	2.297 ± 0.788	2.562 ± 0.915
Posterior Superior Sagittal Sinus	4.230 ± 1.137	4.306 ± 1.126	4.359 ± 1.113	4.259 ± 1.123
Vein of Galen	0.745 ± 0.408	0.741 ± 0.422	0.758 ± 0.419	0.756 ± 0.402
Right Transverse Sinus	4.129 ± 2.230	4.113 ± 2.165	3.565 ± 1.891	4.134 ± 2.218
Left Transverse Sinus	2.521 ± 1.566	2.667 ± 1.640	2.363 ± 1.417	2.632 ± 1.571
**Average Difference Due to Correction**		0.036 ± 0.048	0.088 ± 0.155	-0.017 ± 0.059
**Average Percent Difference**		1.04%	-6.04%	-0.51%
**Average Coefficient of Variation**	0.418	0.457	0.454	0.460

LBC—local bias correction, LPC—local polynomial correction, WBPC—whole brain polynomial correction

## Discussion

This study is one of the first comprehensive investigations of the potential impact of phase-error correction schemes on time-averaged 3D PC imaging of the cerebral vasculature. Previous work in cardiac PC imaging suggested that there may be large measurement errors [3]. Many previous studies predated the now widely-available correction for concomitant gradient effects [14]. In this work, we compared three schemes for correcting eddy current-induced errors in brain PC-MR datasets. Evaluation of phase-error correction schemes can use tissue regions where velocity or VFR is known, such as in stationary background tissue where the flow is expected to be zero cm/s. In this study we showed that in background tissue the absolute VFR was reduced by an average of 57.2% (65.6% with the LBC, 58.4% with the LPC, and 47.7% with the WBFC schemes). LBC showed the largest bias reduction but statistically was not significantly better than LPC or WBPC (*p* = 0.242).

When measuring blood flow in vessels, none of the correction schemes resulted in significant VFR measurement differences (corrected *vs* uncorrected, *p* = 0.997). We, therefore, conclude that the effect of eddy current-induced phase errors in typical 3D PC images of the cerebral vasculature has a negligible impact on VFR measurements. Further, we found no significant difference between correction schemes (*p* = 0.738) in the vessel VFR measurements. While our results are contrary to other reports, those studies imaged near the heart with very different acquisition strategies [2, 10, 15]. However, our study is the first to examine the phase error effects in a typical, current, time-averaged 3D whole-brain PC protocol of the cerebral vasculature.

These results showed that there was slightly more variation in the phase error corrected VFR measurements than in the uncorrected measurements. This manifested as slightly higher coefficient of variation in the corrected vessel measurements. This result is not surprising given the lack of significant bias to correct and the fact that each correction method is sensitive to noise. Without evidence of significantly increased accuracy with these correction schemes, the decreased precision warrants caution in using these phase-error correction schemes.

Other metrics such as wall shear stress, pressure, and kinetic energy [12, 13, 24, 25] would also be impacted by background phase error. Though the analysis of the propagation of errors for these metrics is beyond the scope of this study; some inferences could be made from our findings. We would not expect wall shear stress to be adversely affected by a bias since it is derived from the gradient across the vessel [12]. Pressure and kinetic energy calculations would be more sensitive to a bias error and we would expect error propagation similar to the VFR metrics.

A static phantom was used in these experiments to assert the magnitude of the background phase error. A flow phantom [26, 27] potentially could also be used to assert the accuracy of the flow quantification in the presence of eddy currents, but the precision of the flow phantom would need to be much smaller than the expected bias due to background phase (*i*.*e*., ~<0.2 cm/s).

We have proposed that eddy currents are the primary source of residual phase error, but it needs to be pointed out that the source of these residual errors may also originate from the concomitant phase error not being completely removed or from other sources. When adjusting the velocity encoding value, we found that the bias to be proportional, but this does not preclude other sources of error that might also be present.

While small changes were seen after applying the phase-error correction schemes (on the order of 0.5% to 6%), these changes were much less than the 10% to 25% changes seen in previous cardiac MR-PC imaging studies [2, 3, 8, 9]. The exploration of phase-error correction in cerebrovascular studies, or in time-averaged experiments is not as well represented in the literature, making these findings unique and novel. As stated in some previous reports, phase error may be left uncorrected as the correction may introduce more error than it removes [17]. We can conclude that correcting for phase error in the background tissue did indeed result in a significant effect. However, it can also be concluded from this experiment that there was not a significant difference to the *in vivo* vessel measurements when applying phase error correction.
